# Skin Graft Versus Local Flaps in Management of Post-burn Elbow Contracture

**DOI:** 10.7759/cureus.20768

**Published:** 2021-12-27

**Authors:** Mohamed Issa, Marwa Badawi, George Bisheet, Mahmoud Makram, Abdelhamed Elgadi, Ayyat Abdelaziz, Khaled Noureldin

**Affiliations:** 1 Surgery, Wirral University Teaching Hospital, Wirral, GBR; 2 Surgery, Prince Charles Hospital, Myrther Tydfil, GBR; 3 General Surgery, Conquest Hospital, Hastings, GBR; 4 General Surgery, Cairo University Hospital, Cairo, EGY; 5 Plastic and Reconstructive Surgery, Cairo University, Cairo, EGY; 6 General Surgery, Benghazi Medical Center, Benghazi, LBY; 7 Obstetrics and Gynaecology, Menia University, Menia, EGY; 8 Colorectal Surgery, Southend University Hospital, National Health Service (NHS) Trust, Essex, GBR

**Keywords:** physiotherapy, burn injury, flaps and grafts, split-thickness skin graft (stsg), contracture release, elbow contracture, postburn contracture

## Abstract

Introduction

Contracture is a pathological scar tissue resulting from local skin tissue damage, secondary to different local factors. It can restrict joint mobility, resulting in deformity and disability. This study aimed to investigate the outcomes of skin grafts compared to local flaps to reconstruct post-burn elbow contractures. These parameters included regaining function, range of movement, recurrence, and local wound complications.

Methodology

A retrospective study reviewed 21 patients for elbow reconstruction over 12 months. Only patients with post-burn elbow contracture were included. Other causes, including previous corrective surgery, associated elbow stiffness, and patients who opted out of post-operative physiotherapy, were excluded. Patients were categorized according to the method of coverage into three groups: graft alone (G1), local flap (G2), or combined approach (G3).

Results

Females were three times at higher risk to suffer a burn injury, while almost half of the cases were children. Scald injury represented 81% of burn causes. G1,2,3 were used in 47.6%, 42.9% and 9.5% of cases retrospectively. The overall rate of infection was 28.6%. Hundred percent graft taken was recorded in 83.3 % of cases; however, flap take was 91.1%. After 12 months of follow-up, re-contracture was 60% and 22.8% in G1 and G2; however, the satisfaction rate was 70% and 100% in both groups retrospectively. The overall satisfaction was 85.7% in all groups.

Conclusion

Grafts and local flaps are reasonable options for post contracture release; however, flaps are superior. Coverage selection depends on the lost tissue area and exposure of underlying deep structures. Physiotherapy and patient satisfaction are crucial in the outcomes.

## Introduction

Contracture is a pathological outcome resulting from tissue damage of a large skin surface and resulting in scar formation. The fibrous scar tissue retracts and tethers the surrounding healthy skin, causing skin tightness. This process, in turn, can affect muscle function and joint mobility, resulting in deformity and disability [1].

Elbow joint is a complex joint between the arm and forearm. Its range of movement is 0′-145′ for the extension/flexion motion and 50′-50′ for supination/pronation action. Usually, most activities require a 100′ arc of motion at the elbow to be considered functional. A 30-degree loss of extension can be well managed and compensated. The elbow functions to move, position, and fix the hand in space. Elbow motion is essential to position the hand correctly for daily activities and functions [2].

There are broad causes for elbow contracture/stiffness, which can be extrinsic or intrinsic. The local pathological causes of elbow contracture include; burn, trauma, inflammation, local malignancies, fracture malunion, ligaments contracture, or capsular adhesions [3].

Burn represents the second most common cause of trauma-related deaths in developing countries. An extensive burn can lead to death if not initially managed adequately. However, survival is the immediate concern, restoration of the pre-injury status and return to the routine activities are crucial for the patient and his treating team [4].

Post-burn scarring after deep burn leads to contracture and skin tightness. Pathologically, this can be explained by the over-proliferation of myofibroblasts causing pathological contracture [5].

Management of contracture may improve with time, but it does not resolve on its own. In typical situations, it mandates physiotherapy and/or reconstructive surgery. The main issue that usually concerns surgeons is the methods to cover the raw area after releasing a contracture which varies between skin grafts and different types of local flaps [6].

This study aims to analyse the outcomes of skin grafts compared to local flaps in the reconstruction of post-burn elbow contractures. These parameters included regaining function, range of movement, recurrence, and post-operative local complications, such as infection and graft rejection.

## Materials and methods

A retrospective study reviewed 21 patients admitted for elbow reconstruction in EL Taqwa specialized Hospital over 12 months from September 2017 to September 2018. Only patients with post-burn elbow contracture were included. Elbow contracture for any other reasons, previous corrective surgery, and associated elbow stiffness (pre-operatively) were excluded from this study. Patients who did not receive early post-operative physiotherapy were also excluded. Early post-operative physiotherapy was defined as the therapy starting immediately after four weeks. In this cohort of patients, three operative techniques were used to cover the raw area after releasing the elbow contracture; split-thickness skin graft (STSG) alone, flap, or combined (flap + STSG). Patients who had skin grafts only were categorized as group 1 (G1), patients who had flap only were tagged as group 2 (G2), and group 3 (G3) for the combined approach. The coverage method was based on the intra-operative surgeons' assessment. Flaps were essential for any exposed tendon, nerve, blood vessel, or bone). the use of STSG was either for a superficial raw area or for completion in the areas where the flap coverage was not satisfactory. The hospital records were reviewed to extract the relevant data (clinical letters, operative details, discharge plan, physiotherapy letters and post-operative care) to this work. Ethical approval was obtained locally from EL Taqwa Specialized Hospital's ethical committee.

The preoperative protocol was as follows: (1) History talking: Each patient's medical history was carefully reviewed regarding the time of burn, time of first hospital admission, the physiotherapy patient received afterburn and the history of previous operations. (2) Examination: The patients were assessed regarding (A) the scar's Shape. (B) Type, degree, and severity of the contracture. (C) Elbow joint examination, including the range of movement. (D) Presence of surrounding healthy skin. (E) Vascular and neurological examination of the upper limb. (3) Investigations: (A) Routine blood tests including complete blood counts, coagulation profiles, blood grouping, liver and kidney functions (B) Plain x-ray in two different positions for the elbow region to exclude the preoperative stiffness. (C) Pre- and post-operative photographing of the contracture. 

**Figure 1:**
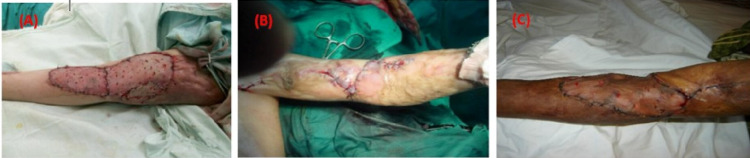
Intra-operative photos for the different surgical techniques (A) Release and application of split-thickness skin graft (STSG) alone. (B) Release and Z-plasty flap alone. (C) Release and Z-plasty flap + STSG.

On reviewing the operative technique, two plastic surgeons were the primary operators. It was as follows: (A) All patients were operated on under general anaesthesia and received preoperative antibiotics according to the local guidelines. (B)Transverse incision at the anterior joint aspect at the joint line level (in one patient, excision of a hypertrophic scar was performed). (C) Gentle extension of the joint up to the entire possible range (all patients achieved extension at 0-30 degrees) followed by proper haemostasis. Three different techniques were used to cover the raw area after the complete release of the contracture; STSG alone, flap alone or flap + STSG (combined) (Figures 1A-1C). The skin graft was taken from the medial aspect of the thigh (the donor site). The graft was fixed using 3/0silk, and a tie-over was applied to fix the graft in place, and the donor site was covered with Vaseline gauze and a pressure bandage. Flaps were indicated to cover exposed tenon, nerve, or blood vessels. Three types of flaps were done; Z-plasty, medial, and lateral arm flaps. STSG was applied in one case with a lateral arm flap and a case with the Z-plasty. An above elbow slab was applied for patients with grafts.

Post-operatively, the first graft dressing was done five days after the operation with normal saline and antibiotic ointment (Garamycine ointment); however, the dressing for patients with only flaps was done after two days. The stitches were removed after two weeks, and the donor site was exposed after three weeks.

The range of movement was measured intra-operatively (after correction of the elbow contracture) and in the follow-up appointments (at three, six, and 12 months' dates). The measurement was as follows; first, the patient was positioned supine with the arm at the side, straightened elbow, and supinated hand. Then a protractor was used to measure the angle between the longitudinal axis of the forearm and the horizon. The range of movement was divided post-operatively into three groups; Group A (0-<30), Group B (30-< 50), and Group C (> 50) degrees.

All patients in this study were subjected to a three-month post-operative physiotherapy programme. This protocol entailed continuous passive movement two times per day while the limb was in a splint, from day 1 post-surgery. The splint was removed after four weeks, and the patient was referred to the hospital physiotherapy unit to start comprehensive physiotherapy three months after slab removal. The follow-up period was at three, six, and 12 months when the range of extension of all patients was measured, and the patient satisfaction status was documented (patient satisfaction was measured as the patient's ability to perform the daily activities according to the patient's routine). On reviewing the follow-up documentation, we found the patient had a different extension range; therefore, data were categorized into three groups; Group A (0-<30), Group B (30-<50), and Group C (>50) degrees, according to the degree of extension.

## Results

Twenty-one patients met our inclusion criteria. Female patients were nearly triple the number of male patients (16 and five retrospectively). The mean age was 23.95 (range 8-38) (Table 1).

**Table 1:** Different age groups of patients

Age groups	Number of patients	Percentage
8-16	10	47.6
18-30	7	33.3
31-38	4	19.1
Total	21	100

**Figure 2:**
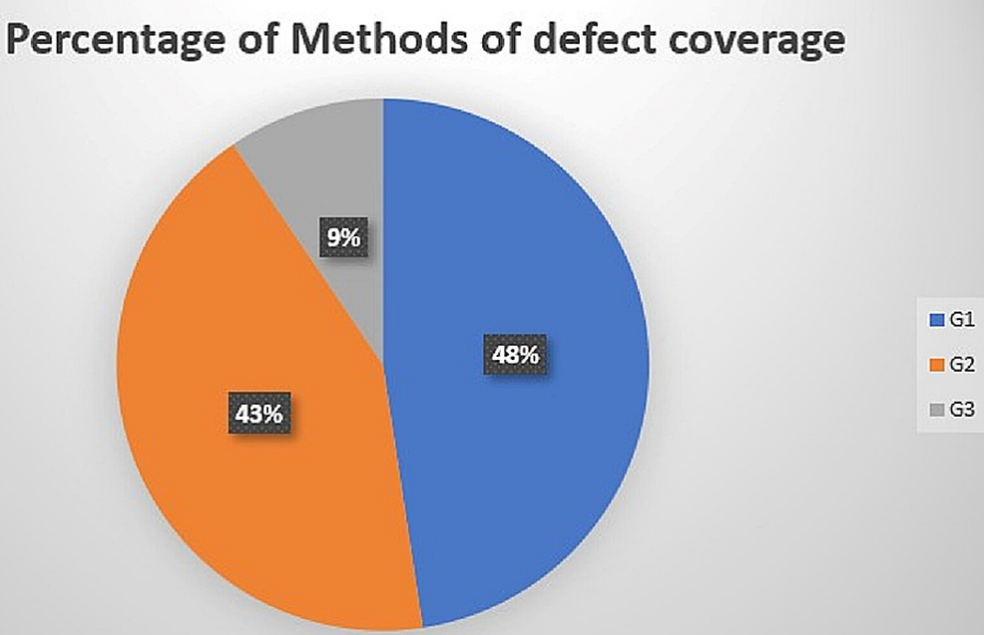
Percentage of methods of the defect coverage G1: Group 1; G2: Group 2; G3: Group 3

The majority of burns,17 patients (81%), were caused by scald (liquid) burn; however, the flame burn was represented by only four cases (19%). STSG was the only way of coverage in 47.6% of cases; however, 42.9% of the patients were reconstructed using the only flap option (Figure 2).

100 per cent of patients with total or medial contracture were treated with STSG only. On the other hand, all patients with linear or strip contracture were treated with release and flap reconstruction; 88.9% had flap only, and the rest had a combined option (11.1%). Different types of contractures and the way of intervention are shown in Table 2.

**Table 2:** Contracture classification and methods of reconstruction STSG: Split-thickness skin graft G1: Group 1; G2: Group 2; G3: Group 3

Type of contracture	Type of intervention			Total no. (%)
	STSG only (G1)	Flap only (G2)	Combined (G3)	
Edge contractures	4	1	1	6 (28.6)
Linear or strip contracture	0	8	1	9 (42.9)
Total contractures	4	0	0	4 (19)
Medial contractures	2	0	0	2 (9.5)
Total No.	10 (47.6%)	9 (42.9%)	2 (9.5%)	21 (100)

**Figure 3:**
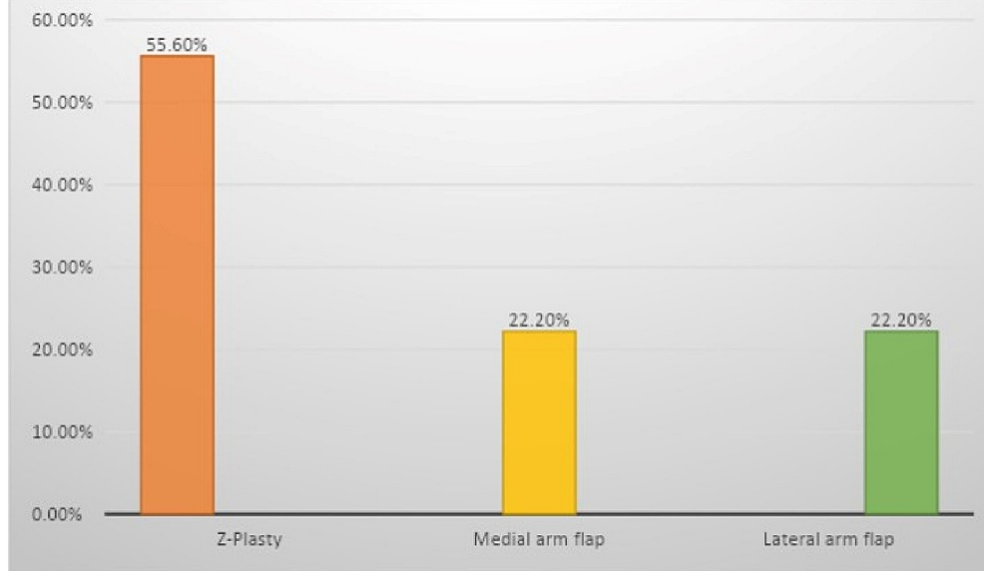
Types of flaps used in reconstruction

Different types of flaps are used to reconstruct the raw area after the release of the contracture (Figure 3). The overall local wound complication was 28.6% (six cases). In G1, two cases (20%) had <15% partial graft loss, while two other cases had mild wound infection. Only one patient (11.1%) had distal flap necrosis in G2. One case in G3 had a mild wound infection. These complications were all treated conservatively with local wound care and oral antibiotics.

At three-month follow-up, 16 (76.2%) of patients had a standard range of extension; however, this percentage decreased to 66.7% and 57.1 % at six and 12 months retrospectively. The rate of patient satisfaction after 12 months of follow-up was 85.7%; however, two patients (9.5%) required re-operation (these two cases developed a re-contracture which affected their basic and essential daily routine; thus, patients were not satisfied with the outcome of their surgery) (Table 3).

**Table 3:** Range of extension and patients’ satisfaction over the follow-up periods STSG: Split-thickness skin graft G1: Group 1; G2: Group 2; G3: Group 3

		Degree of extension			Number of satisfied patients	Total number
		Group A (0-<30^0^)	Group B (30^0^-<50^0^)	Group C (>50^0^)		
Three months	STSG (G1)	6 (60%)	4 (40%)	0	10	10
	Flap only (G2)	9 (100%)	0	0	9	9
	Combined (G3)	1 (50%)	1 (50%)	0	2	2
	Total Number	16 (76.2%)	5 (23.8%)	0	21(100%)	21
Six months	STSG (G1)	5 (50%)	3 (30%)	2 (20%)	9 (90%)	10
	Flap only (G2)	8 (88.9%)	1 (11.1%)	0	9 (100%)	9
	Combined (G3)	1 (50%)	1 (50%)	0	2 (100%)	2
	Total Number	14 (66.7%)	5 (23.8%)	2 (9.5%)	20 (95%)	21
12 months	STSG (G1)	4 (40%)	3 (30%)	3 (30%)	7 (70%)	10
	Flap only (G2)	7 (77.8%)	2 (22.2%)	0	9 (100%)	9
	Combined (G3)	1 (50%)	1 (50%)	0	2 (100%)	2
	Total Number	12 (57.1%)	6 (28.6%)	3 (14.3%)	18 (85.7%)	21

## Discussion

The main target of contracture treatment is to establish an acceptable range of movement to restore patients’ function without causing any pain or instability. Post-burn contractures are distressingly common and severe in developing nations and significant problems in developed countries [2,7].

Different methods have been used to correct this problem. Non-surgical measures, such as splinting and external fixation systems plus physiotherapy can achieve acceptable results. The principle of all surgical interventions is to excise the scar tissue, release the joint and cover the defect with skin grafts, flaps, or combined, according to the depth and size of the raw area and the exposure of the neurovascular structures [8].

Skin grafts and skin flaps are widely used in plastic reconstructive surgeries, especially post-burn scars and deformities. Skin grafts are composed of a thin epidermal layer (outermost layer containing primarily keratocytes and other cells, such as Merkle cells, Langerhans cells, and melanocytes) and a portion of the dermis, which is a fibrous layer formed mainly of collagen and elastin. The skin grafts do not have their blood supply, and they depend on the vascularity of the wound bed to receive their nutrition. Skin grafts take occur through three steps; inhibition, inosculation, and revascularization. Skin grafts are an essential part of the reconstructive ladder. Skin grafts are indicated to cover deep partial-thickness skin defects, full-thickness skin defects when simple measures, such as primary closure and secondary healing, are not feasible. Wounds with active infection and known cancers are an absolute contraindication to skin grafts on top. On the other hand, skin flaps are more useful than grafts on mobile areas, joints, and sphincters. They are considered superior because they do not cause secondary contracture, and they match the same colour and texture of the surroundings [9-11].

Local skin flaps can simply be classified according to sliding, and lifting tissue movements are praised. Flaps include advancement, pivotal, transpositional, rhombic, and bilobed flaps. In this research, advancement flaps were the most popular method to cover the resultant wound bed. These types of flaps have linear or rectangular configurations. They are subdivided into single pedicle, bipedicle, and V-Y flaps. Flaps have reliable perfusion; thus, flaps can be used to cover successfully, relatively avascular areas such as cartilage and bones, with an excellent rapid healing process. In a skilfully implanted skin flap, the rate of complications and morbidity should be equal to those after skin grafting. However, there are potential negative outcomes to consider when choosing skin flaps as a reconstructive modality. Ischemia of the flap is the most feared for all surgeons that can result in a non-viable flap with a large and complex wound, protracted healing course, and a poor aesthetic outcome. Other side effects include failure, haemorrhage, and infection [12,13].

Post-operative physiotherapy should be mandatory to achieve the best outcomes and decrease the rate of failure and recurrence. There are still some arguments about the best regimen to adopt after surgical correction of contracted joints. Continuous passive movement is routinely added as a post-operative program [14]. However, in a study, Morrey used a protocol of continuous passive motion followed by dynamic splinting. Morrey's programme needed a lengthy hospital stay, and later it was changed to three days of passive movement during admission, followed by the dynamic splinting to be started on discharge [15]. Wada et al., in their non RCT, discovered no difference in the results of patients who received post-surgical continuous passive movement, which was supported by Chantelot, who assessed the factors affecting surgical correction to treat elbow scars. A deterioration was observed by another series, the researcher used thermoplastic moulded splint for two weeks, then comprehensive physiotherapy and night splinting for six weeks [16]. Despite that, the final correction was 5′-10′ less than the intra-operative readings, with more decrease in the medium term.

This retrospective study investigated 21 cases with post-burn elbow contracture without any degree of elbow stiffness. Scald burn was the most leading cause for elbow contracture (81%). Contracture in females was threefolds higher than that in males. It is noticed that children were the most affected group in this cohort, with almost half of the set (47.6%) injured, the percentage decreased as the age increased, to reach 19.1% in the eldest age group (30-38 years old). Thus, in this research, it is found that children can suffer domestic elbow injuries that can complicate with elbow disability. In 2019, Mary et al. noticed that flame injury was most popular in adult groups in their study [17]. This can explain the link between the mode of injury with the age group, working-age, and the type of work.

In this study, skin grafts were used in 10 cases (47.6%), while local skin flaps were used in nine cases (42.9%). In two cases, both grafts and local flaps were combined to reconstruct the elbow after excision of the scar tissue. Flaps were used when the raw area was deep, and the neurovascular bundle, tendons, or bone were exposed. Lateral arm flap, medial arm flap, and Z-plasty were used in two, two and five patients, respectively. The take rate for grafts was (100%); however, when looking at the patients who had grafts as coverage (G1 & 3), two cases developed partial graft loss (16.7%). On the other hand, the take of the flaps (in G2 & 3) was (91.1%), as one case developed partial distal necrosis; these partial losses were managed conservatively with the help of tissue viability nurses. Nevertheless, the overall complication rate was 28.6%. The rate of complications was higher in the graft subgroup (four cases). The adverse events were as follow; infection (two cases for grafts and one for combined flap/graft group), bleeding (zero case), a slough of the graft (one case had partial distal necrosis). Serious flap complications, such as flap rejection or ischemia, were not witnessed in this experience. In the following appointments at first, third, and sixth months, and with the physiotherapy protocol, the incidence of overall contracture recurrence was 42.9% (nine cases had post-operative re-contracture >30′). The recurrence rate in the grafted elbows was threefolds higher than the elbows reconstructed with flaps. The incidence of recurrence started to be witnessed in the clinic appointments, after three months of total five cases (G1, 2, 3 were four, zero, one cases retrospectively), seven patients at six months (G1, 2, 3 were five, one, one cases retrospectively), and peaked to nine cases at 12th months (G1, 2, 3 were six, two, one cases retrospectively). The degrees for re-contracture were divided into three groups; A (0-<30), B (30-<50), and C (>50). Patients in group A (57.1%) were reassured and asked to go back to their daily routine as long as their daily activities were not affected. Group B (28.6%) patients were asked to continue a comprehensive physiotherapy course for six months and be reassessed after that period; however, they were satisfied with the final outcome as it did not affect their daily routine. While in group C (14.3%), the patients were not satisfied with the final outcome at 12 months; thus, they had to undergo re-do surgery.

Sanjay [18], in his prospective work, investigated 67 patients with post-burn contracture over 14 months. Similar to this project, Sanjay et al. utilised STSG, and local skin flaps post contracture release. However, the prospective study was broader and included burn contractures at different body areas; head and neck (12 cases), upper limbs (45 cases), lower limbs (nine cases), and body core (one case). Unlike this study, Sanjay found that flame is the most prominent cause of contracture with almost 75%. Flaps were used in less than 30%, while grafts coverage was 73%. The operators used different variants of local flaps; Z-plasty, V-Y plasty, five flap plasty, and seven flap plasty to recontract the wound areas.23.9% developed infections, and 19.5% had minor graft loss. Incidence of recontracture in grafts and flaps were 12% and 0%, respectively. In their result analysis, the range of movement was excellent (44.4%) and good (50%) in the flap group; only one case had poor outcomes. These rates differed in the graft group, three cases had poor outcomes, and a significant number (86%) had a satisfactory range of motion.

Our work, supported by the comparisons of other studies, goes with the literature that skin grafts and flaps are feasible options for post-burn skin contracture to restore elbow function and range of motion. Incidence of rejections, graft loss, and contracture rates are higher in grafts than in flaps. Local skin flaps are reliable for soft tissue replacement. Flaps are classified according to composition, transfer method, shape, and blood supply, which is considered the most vital factor for its outcome [19-21].

A limitation in this study was not including the long-term outcomes (>12 months) for the recurrent contractures, especially those in group C and was scheduled for re-do surgery. Therefore, a different study is recommended with further inclusion and exclusion criteria to concentrate on managing elbow-contracture recurrence.

## Conclusions

According to this experience, scald burn was the most common cause of post-burn elbow contracture, especially in children. Excellent results can be achieved by using either STSG or local flaps. Local flaps are superior to STSG in terms of the outcomes; however, coverage selection depends on the lost tissue area, depth, and the exposure of underlying deep structures. Physiotherapy is recommended to achieve the best results, and patient satisfaction is crucial in the final decision.
